# Genome-Wide Analysis of Fruit Color and Carotenoid Content in *Capsicum* Core Collection

**DOI:** 10.3390/plants13182562

**Published:** 2024-09-12

**Authors:** Nayoung Ro, Hyeonseok Oh, Ho-Cheol Ko, Jungyoon Yi, Young-Wang Na, Mesfin Haile

**Affiliations:** National Agrobiodiversity Center, National Institute of Agricultural Sciences, Rural Development Administration, Jeonju 54874, Republic of Korea; zzjiy@korea.kr (H.O.); hchko@korea.kr (H.-C.K.); naaeskr@korea.kr (J.Y.); ywna@korea.kr (Y.-W.N.)

**Keywords:** *Capsicum*, carotenoids, fruit colors, GBS, GWAS, SNP

## Abstract

This study investigated carotenoid content and fruit color variation in 306 pepper accessions from diverse *Capsicum* species. Red-fruited accessions were predominant (245 accessions), followed by orange (35) and yellow (20). Carotenoid profiles varied significantly across accessions, with capsanthin showing the highest mean concentration (239.12 μg/g), followed by β-cryptoxanthin (63.70 μg/g) and zeaxanthin (63.25 μg/g). Total carotenoid content ranged from 7.09 to 2566.67 μg/g, emphasizing the diversity within the dataset. Correlation analysis revealed complex relationships between carotenoids, with strong positive correlations observed between total carotenoids and capsanthin (r = 0.94 ***), β-cryptoxanthin (r = 0.87 ***), and zeaxanthin (r = 0.84 ***). Principal component analysis (PCA) identified two distinct carotenoid groups, accounting for 67.6% of the total variance. A genome-wide association study (GWAS) identified 91 significant single nucleotide polymorphisms (SNPs) associated with fruit color (15 SNPs) and carotenoid content (76 SNPs). These SNPs were distributed across all chromosomes, with varying numbers on each. Among individual carotenoids, α-carotene was associated with 28 SNPs, while other carotenoids showed different numbers of associated SNPs. Candidate genes encoding diverse proteins were identified near significant SNPs, potentially contributing to fruit color variation and carotenoid accumulation. These included pentatricopeptide repeat-containing proteins, mitochondrial proton/calcium exchangers, E3 ubiquitin-protein ligase SINAT2, histone–lysine N-methyltransferase, sucrose synthase, and various enzymes involved in metabolic processes. Seven SNPs exhibited pleiotropic effects on multiple carotenoids, particularly β-cryptoxanthin and capsanthin. The findings of this study provide insights into the genetic architecture of carotenoid biosynthesis and fruit color in peppers, offering valuable resources for targeted breeding programs aimed at enhancing the nutritional and sensory attributes of pepper varieties.

## 1. Introduction

*Capsicum*, commonly known as pepper, is a globally significant crop valued for its economic importance and nutritional benefits [1]. The genus *Capsicum* includes a diverse range of cultivars, with fruit color and carotenoid content being key traits of interest for both consumers and breeders [2]. The vibrant colors of pepper fruits are primarily determined by the accumulation of various carotenoids, which not only provide visual appeal but also contribute significantly to the fruit’s nutritional value [3]. Peppers are rich in bioactive components and essential nutrients, including vitamins (A, C, and E), colored pigments (zeaxanthin, β-carotene, violaxanthin, capsanthin, β-cryptoxanthin, lutein, and capsorubin), as well as phenolic compounds like capsaicinoids and flavonoids [4,5].

The variations in pigments in pepper fruits reflect combinations of three groups: chlorophylls, carotenoids, and anthocyanins [6,7]. Chlorophylls impart the fruit a green color, whereas the red color is mainly due to carotenoid pigments, such as capsanthin and capsorubin [6,8,9]. Yellow and orange hues primarily result from a varied accumulation of carotenoids, such as violaxanthin, lutein, and *β*-carotene [10]. In contrast, the purple color of unripe fruit arises from anthocyanidins, particularly delphinidin derivatives, in the fruit exocarp [11,12]. When very high levels of anthocyanins, chlorophyll, and some carotenoids coexist in the exocarp, the fruit appears black [13]. The biosynthesis and accumulation of carotenoids in *Capsicum* fruits involve complex metabolic pathways regulated at multiple levels [14].

Carotenoids play a crucial role in human health and nutrition, extending far beyond their function as colorful pigments in fruits and vegetables. These compounds are potent antioxidants that help protect cells from oxidative stress and reduce the risk of chronic diseases [15]. For instance, β-carotene serves as a precursor to vitamin A, essential for vision, immune function, and cell growth [16]. Lutein and zeaxanthin are vital for eye health, particularly in preventing age-related macular degeneration [17]. Lycopene, abundant in red peppers, has been associated with a reduced risk of certain cancers and cardiovascular diseases [18]. Moreover, capsanthin, a carotenoid unique to red peppers, has shown promising anti-inflammatory and anti-cancer properties [19]. The carotenoids found in *Capsicum* species not only contribute to the nutritional value of peppers but also are used to develop functional foods and nutraceutical products to promote human health [20]. The concentration of these compounds in pepper can exhibit significant differences influenced by factors such as pepper genotypes, and cultivation practices [5,21,22].

Understanding the genetic basis of these specific carotenoid contents is crucial for developing improved varieties with enhanced visual appeal and nutritional content. Studies have elucidated the genetic control of fruit color and carotenoid content in *Capsicum*. The Y locus, responsible for red fruit color, was identified as the capsanthin–capsorubin synthase (Ccs) gene [23]. The cl locus, controlling light yellow fruit color, has been associated with a mutation in the phytoene synthase (Psy) gene [24]. The capsanthin–capsorubin synthase (Ccs) gene exhibited polymorphism in PCR patterns within the segregating population [25]. This population originated from a cross between two pepper accessions: cv. msGTY-1 with orange fruits and cv. 277long with red fruits. Analysis revealed a deletion in the Ccs gene’s upstream region in orange-fruited plants [25]. Mutation in the β-carotene hydroxylase gene led to β-carotene buildup and changed pepper fruit color from red to orange [6]. Recent studies further expanded our understanding of carotenoid biosynthesis in peppers. Additionally, the role of a chromoplast-specific lycopene β-cyclase (CYC-B) in controlling β-carotene content was demonstrated [26]. These findings collectively contribute to a more comprehensive understanding of the complex genetic network governing fruit color and carotenoid biosynthesis in *Capsicum*, paving the way for targeted breeding efforts to enhance both the visual and nutritional qualities of peppers. However, further studies exploring more additional genes that contribute to fruit color and carotenoid production are important.

Genome-wide association study (GWAS) has emerged as a powerful tool for dissecting complex traits in *Capsicum* and other crop species. In *Capsicum*, GWAS was successfully applied to investigate various traits, including fruit-related characteristics, disease resistance, and metabolite content [27,28,29,30]. This approach has led to the identification of novel loci associated with important agronomic traits, providing valuable insights for pepper breeding programs. The applicability of GWAS extends beyond *Capsicum*, with successful implementations in other crops such as rice, maize, wheat, and tomato [31,32,33,34]. In these crops, GWAS has facilitated the discovery of genetic variants associated with yield components, quality traits, and stress tolerance. The success of GWAS across different plant species demonstrates its versatility and effectiveness in unraveling the genetic architecture of complex traits. Therefore, using a comprehensive genome-wide approach on a diverse core collection, this study aimed to provide additional insights into the genetic control of fruit color and carotenoid content in *Capsicum*.

## 2. Results

### 2.1. Fruit Colors and Carotenoid Content Variations

**Figure 1:**
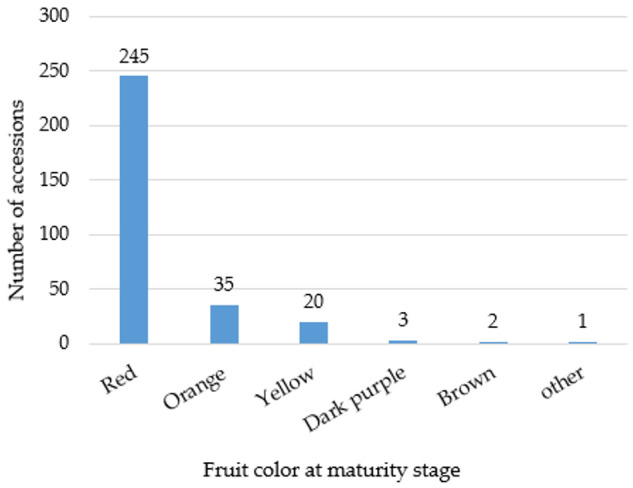
The distribution of 306 pepper accessions based on fruit color at maturity.

**Figure 2:**
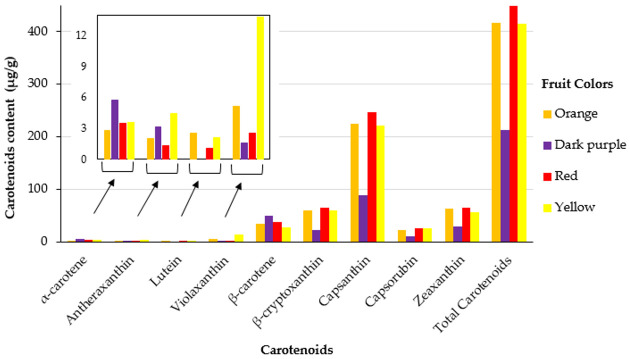
The average carotenoid contents of pepper accessions based on fruit color.

**Figure 3:**
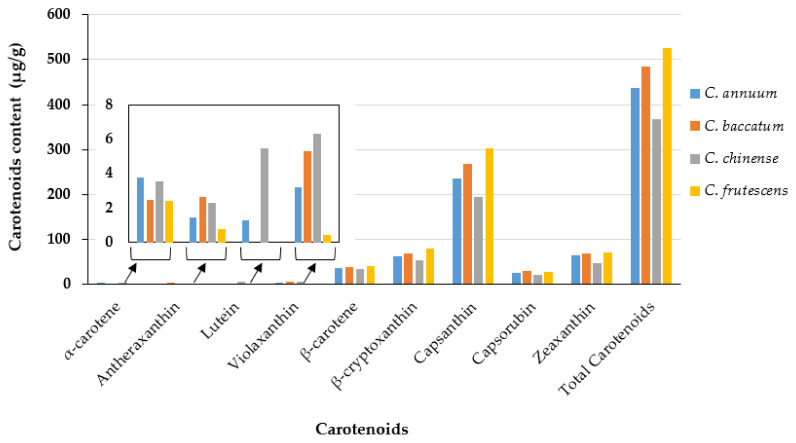
The average carotenoid contents of pepper accessions based on species. These accessions represent five species: *C. annuum* (198), *C. baccatum* (43), *C. chinense* (43), and *C. frutescens* (21).

This study incorporated a total of 306 pepper accessions from diverse *Capsicum* species. The fruit color distribution among the pepper accessions is visualized in Figure 1. Red fruits were the most abundant, with 245 accessions. Orange peppers were the second most common, with 35 accessions, followed by yellow peppers, which had 20 accessions. Fruits with purple, brown, and other uncommon colors were rare, with only three accessions exhibiting dark purple and two accessions displaying brown. The descriptive statistics summary of carotenoid contents across 306 pepper accessions reveals the variability and distribution of these compounds (Table 1). The mean concentrations of the individual and total carotenoids exhibited significant diversity across the dataset. Among the individual carotenoids, capsanthin showed the highest mean concentration, followed by β-cryptoxanthin and zeaxanthin. The descriptive statistics also highlighted the variability within the dataset, with ranges extending from 7.09 to 2566.67 μg/g for total carotenoids. This diversity emphasizes the complexity and richness of carotenoid profiles within the pepper accessions studied. Analysis of carotenoid content in mature *Capsicum* fruits of different colors revealed variations in both capsanthin levels and total carotenoid content. Capsanthin, the predominant individual carotenoid analyzed, showed marked differences across fruit colors (Figure 2). Red *Capsicum* fruits exhibited the highest capsanthin content, followed closely by orange fruits and yellow fruits. The average carotenoid contents of pepper accessions, categorized by species, are summarized in Figure 3. Among the *Capsicum* species, *C. frutescens* exhibited the highest total carotenoid content (526.19 μg/g). Also, β-cryptoxanthin and capsanthin showing notable levels in subsequent order. *C. baccatum* had a total carotenoid content of 484.08 μg/g, characterized by elevated concentrations of capsanthin and zeaxanthin. *C. annuum* and *C. chinense* had lower total carotenoid contents, with *C. annuum* showing notable capsanthin levels. These variations may have been influenced by the differing number of accessions sampled for each species and fruit colors.

### 2.2. Correlation and Principal Components Analysis

**Figure 4:**
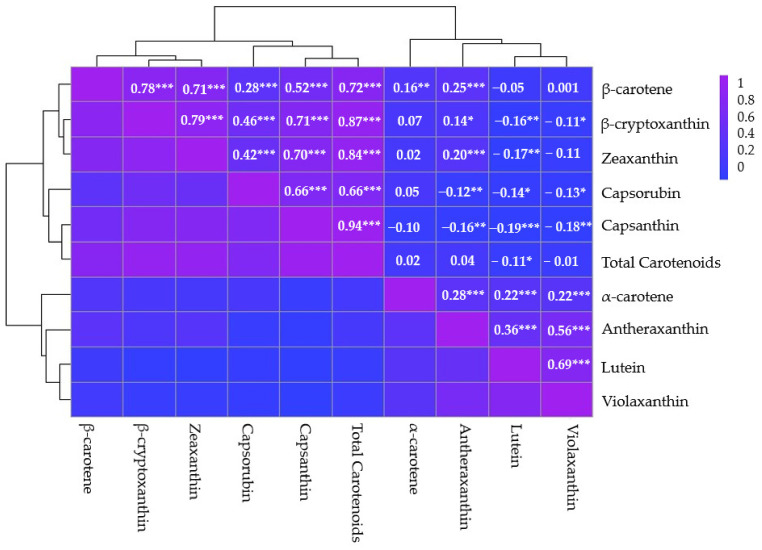
Heatmap depicting carotenoid correlations in a diverse set of 306 pepper accessions. This visualization presents Pearson correlation coefficients, with a color scale on the right indicating correlation strength and direction. The analysis includes the following carotenoids: violaxanthin, lutein, antheraxanthin, capsorubin, capsanthin, zeaxanthin, β-cryptoxanthin, α-carotene, β-carotene and total carotenoid content. Significance is represented by *, **, and *** for *p*-values of less than 0.05, 0.01, and 0.001, respectively.

Analysis of the correlation matrix among carotenoid compounds in pepper accessions provided valuable insights into the interrelationships between these bioactive compounds (Figure 4). Correlation coefficients revealed varying strengths and directions of associations between carotenoid pairs. For instance, a moderate positive correlation (r = 0.560) was observed between antheraxanthin and violaxanthin, suggesting that increases in the concentration of one are associated with increases in the concentration of the other. A strong positive correlation (r = 0.78 ***) between β-carotene and β-cryptoxanthin indicated a close association in their accumulation patterns. In contrast, some carotenoid pairs displayed weaker or negative correlations, such as capsanthin and lutein (r = −0.19 ***), suggesting an inverse relationship. Total carotenoid content exhibited strong positive correlations with several individual compounds, including capsanthin (r = 0.94 ***), β-cryptoxanthin (r = 0.87 ***), and zeaxanthin (r = 0.84 ***). These correlations suggest that these compounds significantly contribute to the overall carotenoid profile in peppers. The observed correlations offer insights into the complex dynamics of carotenoid accumulation in pepper varieties and may inform breeding strategies for enhanced nutritional and sensory attributes.

**Figure 5:**
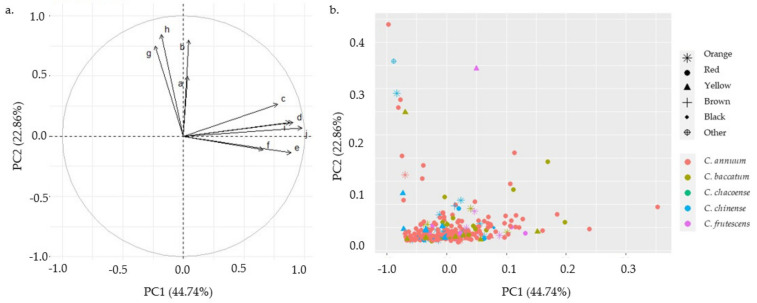
PCA plot based on the carotenoid data of 306 pepper accessions: (**a**)—variables and (**b**)—individuals; each dot represents a single accession. The variables are a—α-carotene, b—antheraxanthin, c—β-carotene, d—β-cryptoxanthin, e—capsanthin, f—capsorubin, g—lutein, h—violaxanthin, i—zeaxanthin, and j—total carotenoid.

PCA was performed on individual and total carotenoid content data from 306 pepper accessions (Figure 5). The analysis accounted for 67.6% of the total variance. Principal Component 1 (PC1) and Principal Component 2 (PC2) explained 44.7% and 22.9% of the variance, respectively. The PCA results confirmed the clustering patterns observed in the correlation heatmap (Figure 5), revealing two distinct carotenoid groups. The first group included α-carotene, violaxanthin, antheraxanthin, and zeaxanthin, while the second group comprised capsorubin, capsanthin, zeaxanthin, β-cryptoxanthin, β-carotene, and total carotenoids. This grouping pattern provides additional evidence of the relationships between specific carotenoids in pepper accessions.

### 2.3. Genome-Wide Association Analysis

**Figure 6:**
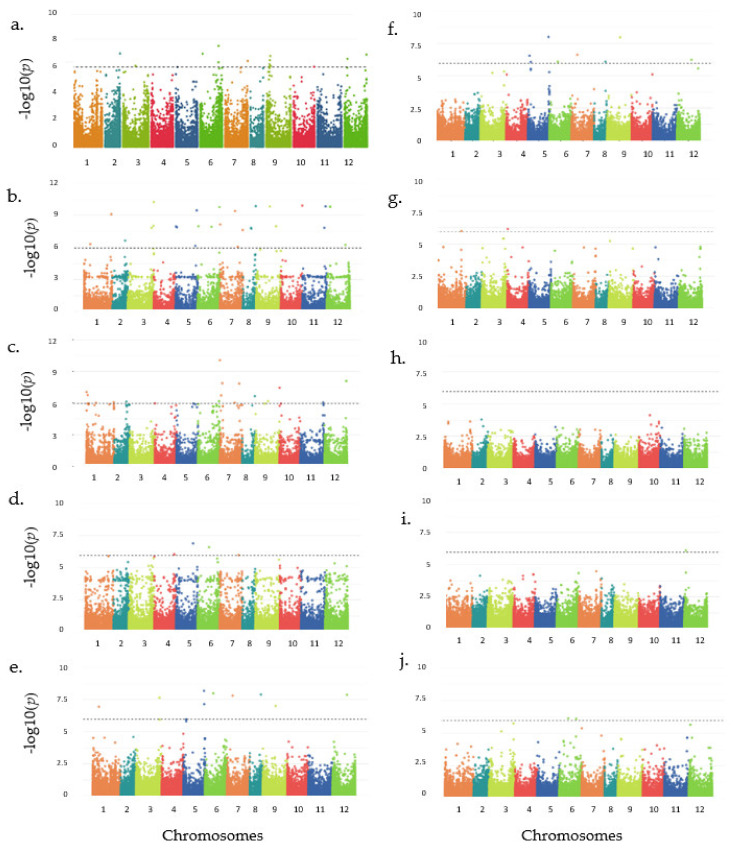
GWAS results for fruit color and carotenoid traits presented as Manhattan plots. The figure comprises plots for fruit color (**a**) and carotenoids: (**b**–**j**) α-carotene, antheraxanthin, β-carotene, β-cryptoxanthin, capsanthin, capsorubin, violaxanthin, zeaxanthin, and total carotenoids. In each plot, SNPs are represented as individual points, with chromosomes distinctly colored and labeled on the *x*-axis. The *y*-axis represents association strength as −log10(p). A grey dashed line at −log10(p) = 6.0 denotes the significance threshold (*p* < 0.05).

Association analysis was conducted to identify SNPs associated with fruit color and carotenoid content in peppers. The distribution patterns of SNPs across the 12 chromosomes, visualized in 1 Mb windows, are shown in Supplementary Figure S1. The association analysis revealed 91 significant SNPs: 15 linked to fruit color (10 genic, 5 intergenic) and 76 to carotenoid content (48 genic, 28 intergenic) (Table 2). Detailed information on all SNPs associated with carotenoids is provided in Supplementary Table S2. The GWAS results are visualized using Manhattan plots (Figure 6) and quantile–quantile (Q-Q) plots (Supplementary Figure S2). Among the carotenoids, α-carotene had the highest number of associated SNPs (28), followed by antheraxanthin (19), β-cryptoxanthin (9), and capsanthin (8). β-carotene was associated with seven SNPs, capsorubin and total carotenoid content had two each, and zeaxanthin had the fewest with one SNP. The significantly associated SNP distribution varied by chromosome as presented in Table 2. Analysis of the genomic regions surrounding significant SNPs associated with fruit color identified several candidate genes (Table 3). These genes encode a variety of proteins including the following: a pentatricopeptide repeat-containing protein At5g66520-like, a RING-type domain-containing protein, a PITH domain-containing protein, three instances of mitochondrial proton/calcium exchanger proteins, tropinone reductase 2, an upstream gene encoding gamete expressed protein 1, beta carbonic anhydrase 5 (chloroplastic), and a putative glutathione S-transferase. The list of significantly associated SNPs with carotenoid content, along with the genes where these SNPs are located, is summarized in Table 4. These genes encode proteins such as E3 ubiquitin-protein ligase SINAT2, sucrose synthase 6, pentatricopeptide repeat-containing protein (chloroplastic), transcription termination factor MTEF18, beta-glucosidase BoGH3B, a probably inactive leucine-rich repeat receptor-like protein kinase IMK2, lignin-forming anionic peroxidase, receptor-like protein 19, and histone–lysine N-methyltransferase ASHH2. These proteins represent diverse cellular functions, including protein degradation, carbohydrate metabolism, chloroplast function, transcription regulation, and various enzymatic activities. SNPs that exhibited significant associations with multiple carotenoid traits, suggesting possible pleiotropic effects, are found in Table 4. One SNP on chromosome 1 was linked to α-carotene and β-cryptoxanthin, while the remaining six SNPs on chromosomes 5, 6, 7, 8, 9, and 12 were associated with β-cryptoxanthin and capsanthin. The genes listed include an E3 ubiquitin-protein ligase, stamen-specific protein, RNA helicase, protein homolog, protein-tyrosine-phosphatase, and a guanine nucleotide exchange factor.

## 3. Discussion

Peppers show remarkable genetic diversity, leading to a wide range of fruit colors, shapes, and sizes. This diversity arises from natural variation, selective breeding efforts, mutations, and environmental influences over generations. Fruit color is a key trait that breeders have targeted to develop new varieties with diverse visual appeal and improved nutritional profiles. Variation in fruit color among pepper accessions is primarily due to differences in the accumulation and composition of pigments. In this study, fruit color distribution analysis among the 306 pepper accessions showed that red fruits were the most abundant, followed by orange and yellow. Red and yellow peppers are rich in carotenoids, contributing to their vivid colors [35]. Carotenoids contribute to the diverse colors of pepper fruits and provide various health benefits, such as acting as dietary sources of provitamin A and protecting against cardiovascular diseases and certain cancers [36,37]. Among the 306 accessions, dark purple and brown fruit colors were rare, with only a few accessions exhibiting these colors. These rare accessions are important because breeders have increasingly focused on developing pepper varieties with diverse fruit colors, such as white, purple, yellow, black, and orange, to meet consumer preferences and enhance the diversity of available pepper commodities [38]. The accumulation and proportion of pigments like chlorophylls, anthocyanins, and carotenoids, determine pepper fruit color [39,40,41,42]. Understanding the genetic basis of fruit color variation is crucial for breeding programs for developing pepper varieties with desirable color traits and nutritional properties.

The study revealed significant diversity in carotenoid profiles across 306 pepper accessions, highlighting the complexity of carotenoid composition in peppers. This diversity is likely attributed to genetic differences among accessions [43]. Capsanthin was identified as the predominant carotenoid, significantly contributing to the total carotenoid content. This finding is consistent with previous reports on *C. chinense* [43,44] and sweet red pepper (*C. annuum*) varieties [45]. In this study, capsanthin contributed an average of 54% to the total carotenoid content, which falls within the range reported in previous studies: 45% in *C. chinense* [44] and 60% as reported by Bosland [46]. Capsanthin is known for its potential health benefits [47,48,49]. Correlation analysis provided insights into the interplay between carotenoid compounds. Strong positive correlations were observed between several carotenoid pairs, including antheraxanthin and violaxanthin, β-carotene and β-cryptoxanthin, and total carotenoids with β-cryptoxanthin, capsanthin, and zeaxanthin. These correlations suggest that changes in one carotenoid’s concentration may correspond with changes in others, underscoring the complex dynamics of carotenoid accumulation within pepper varieties [49].

The number of SNPs associated with fruit colors and carotenoid contents are summarized in Table 3 and Table 4. A total of 15 SNPs significantly associated with fruit color were identified. Among these, some SNPs were located within genes, while others were in the intergenic regions. The genes where the significantly associated SNPs are located include beta carbonic anhydrase 5, chloroplastic, putative glutathione S-transferase, and pentatricopeptide repeat-containing protein (PPR). The association of PPR genes with fruit color traits is consistent with findings from various studies across different plant species. For instance, a genome-wide study in watermelon revealed that SNPs in four PPR genes were significantly correlated with flesh color variation [50]. Similarly, a recent genetic mapping study in watermelon identified a PPR gene co-segregating with the C2 locus, which controls yellow flesh color [51]. These findings suggest a conserved role for PPR genes in fruit color determination across cucurbit species. The involvement of PPR genes in fruit color regulation extends beyond the *Solanaceae* and *Cucurbitaceae* families. In melon (*Cucumis melo*), a QTL analysis identified CmPPR1 as a candidate gene for flesh color phenotype, representing one of two significant loci controlling this trait [52]. Furthermore, in tomato, reduced expression of a PPR-encoding gene was observed in the cnr (colorless non-ripening) mutant, which exhibits colorless pericarp in mature fruits [53]. While the exact mechanism linking PPR proteins to fruit color remains unclear, these proteins are known to influence organellar mRNA transcripts and regulate chloroplast size and thylakoid membrane structure [54,55]. PPR proteins have also been implicated in plastid-to-nucleus retrograde signaling, which can affect the expression of genes targeted to plastids, potentially regulating color variation in a quantitative manner [52,55]. Additionally, PPRs play a role in various RNA modifications, including editing, stabilization, and splicing [56]. A recent study on *C. chinense* further supports the importance of PPR genes in fruit color determination [57]. Their analysis of gene expression profiles revealed varied expression patterns of eight PPR genes across three *C. chinense* accessions with different fruit colors. Interestingly, four PPR genes were significantly upregulated in the red-fruited ‘Naga Morich’ cultivar, while the other four were downregulated, suggesting a complex regulatory network involving PPR genes in fruit color development.

One SNP associated with fruit color was identified in a gene encoding a putative glutathione S-transferase (GST) gene. This finding aligns with recent research on the GST role in anthocyanin transport and fruit pigmentation. Anthocyanins, water-soluble flavonoid pigments, are crucial for fruit color and have additional roles in plant defense and human health benefits [58,59,60]. While anthocyanin biosynthesis is well-understood [61,62], their transport to vacuoles is crucial for fruit coloration. GSTs, particularly the plant-specific Phi class, are involved in anthocyanin transport across various plant species [63]. Loss of GST function reduces anthocyanin accumulation in plants such as maize, petunia, and Arabidopsis [63,64,65,66]. In fruit crops, several GSTs are associated to pigmentation, including LcGST4 in litchi, VviGST4 in grapevine, RAP in strawberry, MdGSTF6 in apple, and PpGST1 in peach [67,68,69,70,71]. The SNP identified could affect GST function or expression, potentially influencing anthocyanin transport and fruit color. This is similar to the effects observed with the Bronze-2 (Bz2) GST gene knockout in maize [64].

Among the SNPs associated with carotenoids, we identified variants in genes encoding functional proteins such as E3 ubiquitin-protein ligase SINAT2, histone–lysine N-methyltransferase, protein SCAR1 (AtSCAR1), sucrose synthase 6 (AtSUS6), DENN domain and WD repeat-containing protein SCD1, hexokinase-1, putative disease resistance protein RGA1 (RGA3-blb), ribosomal biogenesis protein LAS1L, and 3-oxoacyl-[acyl-carrier-protein] synthase (mitochondrial). Of particular interest, an SNP on chromosome 1 (69188981 bp) associated with carotenoid content was found within a gene encoding an E3 ubiquitin-protein ligase. The association between E3 ubiquitin-protein ligases and carotenoid content is consistent with the known regulatory roles of these enzymes in plants. A previous study showed that SINAT2, a SINA-type E3 ligase in Arabidopsis, interacts with the transcription factor AtRAP2.2 to regulate carotenogenesis [72]. This SNP may represent a similar regulatory mechanism, potentially affecting key factors in the carotenoid biosynthetic pathway. SINA E3 ligases are involved in various aspects of plant development, including auxin signaling and lateral root development [73], flowering time regulation [74,75], and brassinosteroid signal transduction [76]. In tomato, SlSINA2 regulates shoot growth and leaf chlorophyll content [77]. These diverse functions highlight the potential interconnectedness of carotenoid metabolism with broader regulatory networks controlling plant growth and development. Findings on E3 ubiquitin-protein ligases suggest potential targets for breeding programs or genetic engineering to modify carotenoid content in plants. Further functional characterization is needed to confirm the direct role of these proteins in carotenoid regulation. The identification of SNPs in genes encoding various functional proteins, especially the E3 ubiquitin-protein ligase, associated with carotenoid content adds to the growing evidence linking these enzymes to carotenoid metabolism [78,79]. These results offer new insights into the regulation of carotenoid biosynthesis and open avenues for crop improvement strategies, focusing on nutritional quality and plant stress responses.

The GWAS results in *Capsicum* revealed an SNP associated with total carotenoid content in a gene encoding histone–lysine N-methyltransferase, contributing to the growing evidence of epigenetic regulation in carotenoid biosynthesis across plant species. This finding aligns with recent research in citrus, which identified CgSDG40, a novel histone methyltransferase gene, as a positive regulator of carotenoid biosynthesis [80]. Histone methylation’s role in carotenoid regulation seems to be conserved across plant species, as initially reported in Arabidopsis [81]. The conservation of these regulatory mechanisms is supported by findings that the genomic arrangement of SDG40 and PSY1 is conserved in Citrus and other plants [80]. Our findings, along with recent studies in Citrus and Arabidopsis, enhance the understanding of epigenetic regulation of carotenoid biosynthesis across plant species. This may lead to more comprehensive models of carotenoid regulation and inform breeding strategies for improved carotenoid content in crops. This study identified a significantly associated SNP with α-carotene levels in pentatricopeptide repeat-containing proteins (PRPs). Carotenoids, including α-carotene, are key pigments responsible for yellow to red coloration in fruits and vegetables. This PRP-carotenoid association is consistent with the identification of a PRP-encoding gene linked to yellow flesh in watermelon [82]. These findings extend previous research by linking PRPs to α-carotene, suggesting that PRPs may regulate particular steps in carotenoid biosynthesis across species. This association implies that PRPs influence fruit pigmentation through modulation of the carotenoid pathway, supported by previous studies demonstrating how genetic variations affect carotenoid accumulation and fruit color [83]. Additionally, the analysis revealed several SNP markers with pleiotropic effects on carotenoid traits, specifically α-carotene, β-cryptoxanthin, and capsanthin levels (Table 4). We identified one SNP associated with both α-carotene and β-cryptoxanthin, and six SNPs associated with β-cryptoxanthin and capsanthin. These markers were primarily found in genes, with associated proteins including E3 ubiquitin-protein ligase SINAT2, stamen-specific protein FIL1, and DEAD-box ATP-dependent RNA helicase 8, among others. The pleiotropic nature of these SNPs indicates shared genetic control mechanisms for multiple carotenoid traits, potentially involving biosynthesis regulation or interactions between metabolic pathways. These findings offer valuable insights into the genetic architecture of carotenoid accumulation and may guide future breeding strategies for modifying carotenoid profiles in plants.

## 4. Materials and Methods

### 4.1. Chemicals and Plant Material

For this study, high-purity reagents, extraction solvents, and carotenoid standards were employed. Chemical compounds were obtained from Sigma-Aldrich, including carotenoid reference materials such as zeaxanthin, β-carotene, capsanthin, violaxanthin, α-carotene, antheraxanthin, β-cryptoxanthin, and capsorubin. Additional reagents included ammonium acetate, ascorbic acid, dichloromethane, methanol, methyl tert-butyl ether, potassium hydroxide, and sodium chloride.

The study utilized 306 pepper accessions obtained from the gene bank of the National Agrobiodiversity Center (NAC) under the Rural Development Administration (RDA), Jeonju, Republic of Korea. These accessions represent five species: *C. annuum* (198 accessions), *C. baccatum* (43), *C. chinense* (43), *C. frutescens* (21), and *C. chacoense* (1). The plants were grown in the RDA research field (35°49′52.7″ N, 127°3′43.9″ E) in a greenhouse from March to October (2020), using standard agronomic practices according to RDA cultivation methods. From March to April, seedlings were prepared, and the temperature was maintained in the range 15–25 °C. In May, the seedlings were transplanted to the soil and cultivated until late October. During this period, the temperature inside the greenhouse was maintained in the range 15–40 °C, and irrigation was performed once or twice a week depending on plant conditions. Each accession consisted of ten pepper plants. Additional information regarding the accession numbers and the origins of the 306 pepper accessions is available in Supplementary Table S1.

### 4.2. Pepper Fruit Color Assessment and Analysis of Carotenoids

Visual assessment of pepper fruit color was performed at maturity on ten plants per accession. Due to variability in maturity time among accessions, regular assessments were carried out to monitor and determine the optimum maturity stage for each plant. The color of each fruit was recorded using predefined categories such as yellow, orange, red, dark purple, brown, and other, based on a standard color chart (QPcard) to ensure consistent evaluation. Examples of different fruit colors observed in pepper accessions are presented in Supplementary Figure S3.

This research utilized freeze-dried, powdered pepper samples for carotenoid analysis. The extraction, separation, and quantification of carotenoids were performed using a modified version of the protocol described by Kim et al. [84]. For extraction, 0.05 g of finely sieved pepper powder was combined with 3 mL of ethanol containing 0.1% (*w/v*) ascorbic acid. After briefly vortexing, the mixture was heated in an 85 °C water bath for 5 min. Saponification was then carried out for 10 min at 85 °C using 120 μL of 80% (*w/v*) potassium hydroxide. Following ice cooling, 1.5 mL of cold deionized water was added. The extraction was repeated twice with 1.5 mL of hexane. The extracts were centrifuged at 12,009× *g*, and the supernatant was filtered through a 0.2 μm syringe filter for analysis.

Carotenoid separation was achieved using an Agilent 1260/90 Infinity II High-Performance Liquid Chromatography (HPLC; Santa Clara, CA, USA) system equipped with a C30 YMC column (250 × 4.6 mm, 3 μm; Waters Corporation, Milford, MA, USA). Detection was performed at 450 nm. The mobile phase consisted of Solvent A (methanol:water, 92:8 *v*/*v*, with 10 mM ammonium acetate) and Solvent B (pure methyl tert-butyl ether). The gradient elution profile was set as follows: 0 min (83% A, 17% B), 23 min (70% A, 30% B), 29 min (59% A, 41% B), 35 min (30% A, 70% B), 40 min (30% A, 70% B), 44 min (83% A, 17% B), and 55 min (83% A, 17% B), with a flow rate of 1 mL/min. Quantification was performed using calibration curves constructed from the peak area ratios of four different concentrations of carotenoid standards.

### 4.3. Genomic DNA (gDNA) Extraction

Leaf samples from 306 pepper accessions were used to extract genomic DNA, employing a modified CTAB protocol based on the method outlined by Lee et al. [85]. The extracted DNA was initially diluted to 50 ng/μL with distilled water. Quantification was performed using the Quant-iT PicoGreen dsDNA Assay Kit in conjunction with a Synergy HTX Multi-Mode Reader. Subsequently, the DNA concentration was adjusted to a standard 12.5 ng/μL. The quantified DNA samples were then subjected to enzymatic digestion using ApeKI (New England Biolabs, Ipswich, MA, USA) for a duration of 3 h at 75 °C.

### 4.4. Library Preparation for Genotyping-by-Sequencing (GBS)

GBS libraries were prepared according to the methods described in previous studies [86,87], with minor modifications. After restriction digestion, the DNA fragments were ligated with adapters, including barcoded adapters for sample identification and common adapters, using T4 DNA ligase (New England Biolabs) at 22 °C for 2 h. The ligase was then inactivated by heating at 65 °C for 20 min. The adapter-ligated samples were pooled and purified using the NucleoSpin^®^ Gel and PCR Clean-up Kit (Macherey-Nagel GmbH & Co. KG, Düren, Nordrhein-Westfalen, Germany). The pooled ligation products were amplified by multiplexing PCR in a 50 μL reaction volume using AccuPower Pfu PCR Premix (Bioneer) and the provided primers. The fragment size distribution of the PCR products was assessed using the BioAnalyzer 2100 (Agilent Technologies, Santa Clara, CA, USA). The GBS libraries were then sequenced on the Illumina NextSeq500 platform (Illumina, San Diego, CA, USA), generating 150 bp single-end reads.

### 4.5. SNP Calling, Filtering, and Sequence Preprocessing

The generated read sequences underwent preprocessing using Stacks [88] for demultiplexing, FastQC [89] for per-base read quality assessment, and Cutadapt [90] for adapter sequence removal. The reads were then aligned to the CM334 reference genome (*C. annuum* chromosome v1.6) using Bowtie2. To facilitate data integration into the GATK pipeline, read groups were added using Picard tools. Localized read realignments were performed using GATK’s ‘IndelRealigner’ and ‘RealignerTargetCreator’ arguments to correct misalignments caused by indels.

Initial variant calling was performed using GATK’s “HaplotypeCaller” and “SelectVariants” parameters. Variants were filtered using GATK’s “FilterVariant” module based on quality score (QUAL < 30), quality depth (QD < 5), and Fisher score (FS > 200). Further filtering was performed using vcftools (v. 0.1.15) to impose restrictions on maximum missing rate (--max-missing 0.95), minimum minor allele frequency (--maf 0.05), allele range (--min-alleles 2, --max-alleles 2), and average read depth (--min-meanDP 5). These steps aimed to identify high-quality SNPs for subsequent analysis.

### 4.6. Genome-Wide Association Study (GWAS)

Genome-wide association study (GWAS) was performed on 306 pepper individuals using 42,322 SNPs, employing TASSEL v.5.0 standalone software [91]. A mixed linear model (MLM) incorporating both population structure and kinship (PCA+K) was utilized. The kinship (K) matrix was derived from an identical-by-state (IBS) matrix, reflecting familial relatedness between lines. A significance threshold of –log10(p) > 6.0 was established after Bonferroni correction.

To identify potential candidate genes associated with significant SNPs, we examined a 200 kb region (100 kb on each side) surrounding each SNP. The basic local alignment search tool (BLAST) was used to search the *C. annuum* genome in both the Ensemble Plants genome database (https://plants.ensembl.org/Capsicum_annuum/Tools/Blast accessed on 7 May 2024) and the NCBI database. Flanking sequences of the significant SNPs were extracted from the *C. annuum* genome database and analyzed to identify genes or gene regions with alignment similarity.

### 4.7. Statistical Analysis

Data summary and descriptive statistics for the carotenoids were conducted using Microsoft Excel 2016 (Microsoft Corporation, Redmond, WA, USA). Correlation analysis (using the ‘pheatmap’ package) and principal components analysis were performed with R software (version 4.2.1).

## 5. Conclusions

This study of 306 pepper accessions revealed substantial diversity in fruit color and carotenoid profiles across *Capsicum* species. Pepper accessions with red fruits were predominant, with capsanthin identified as the primary carotenoid. Correlation and principal component analyses uncovered complex relationships among carotenoids. GWAS identified 91 significant SNPs associated with fruit color and carotenoid content, implicating several candidate genes involved in diverse cellular functions. Notably, seven SNPs exhibited pleiotropic effects on multiple carotenoid traits. Among the carotenoid-associated SNPs, some were located in genes encoding functional proteins such as E3 ubiquitin-protein ligases and histone–lysine N-methyltransferase, which have recently been identified as novel contributors to carotenoid biosynthesis in Citrus. These findings provide valuable insights into the genetic basis of fruit color and carotenoid accumulation in peppers, offering a foundation for future breeding programs aimed at enhancing both the nutritional quality and visual appeal of *Capsicum* varieties.

## Figures and Tables

**Table 1:** Descriptive statistics summary of carotenoid contents (μg/g) of 306 pepper accessions.

Carotenoid	Mean	SE	SD	Minimum	Maximum	No. of Accessions with Measurable Pigment
α-carotene	3.49	0.42	7.36	0	86.94	294
Antheraxanthin	1.71	0.29	5.04	0	53.56	66
β-carotene	36.62	2.22	38.88	0	272.15	304
β-cryptoxanthin	63.7	3.66	64.06	0	476.23	296
Capsanthin	239.12	11.79	206.22	0	1348.53	291
Capsorubin	25.8	1.75	30.57	0	265.99	289
Lutein	1.63	0.58	10.1	0	113.92	12
Violaxanthin	3.76	1.1	19.17	0	182.58	106
Zeaxanthin	63.25	3.36	58.76	0	352.25	284
Total Carotenoid	439.06	19.75	345.52	7.09	2566.67	306

**Table 2:** The number of significantly associated SNPs with fruit color and carotenoids across chromosomes.

Chromosome	Genic	Intergenic	Number of SNPs
1	2	6	8
2	2	1	3
3	4	2	6
4	2	4	6
5	6	4	10
6	12	1	13
7	7	6	13
8	5	1	6
9	4	5	9
10	2	0	2
11	2	1	3
12	10	2	12
Total	58	33	91

**Table 3:** Key SNPs showing significant associations with fruit color in a diverse pepper (*Capsicum* spp.) germplasm collection.

Trait	Chr.	Pos.	−log *(p*-Value)	Ref.	Alt	SNP Type	Protein	Minor Allele	Major Allele
Fruit color at mature stage	2	167079497	6.66	T	C	Genic	RING-type domain-containing protein	C	T
	3	137561221	6.00	G	T	Intergenic	Intergenic	T	G
	3	137561156	6.04	A	G	Intergenic	Intergenic	G	A
	6	200892457	7.20	A	T	Genic	Pentatricopeptide repeat-containing protein	T	A
	6	25789177	6.64	G	A	Genic	PITH domain-containing protein	A	G
	6	200623395	6.03	C	T	Intergenic	Intergenic	T	C
	7	250212419	6.13	G	A	Intergenic	Intergenic	A	G
	9	46177512	6.48	T	A	Genic	Tropinone reductase 2	A	T
	9	41391568	6.18	T	C	Genic	Beta carbonic anhydrase 5, chloroplastic	C	T
	9	37619422	6.02	C	T	Intergenic	Intergenic	T	C
	9	51246107	6.04	C	T	Genic	Putative glutathione S-transferase	T	C
	12	243316298	6.58	T	G	Genic	Mitochondrial proton/calcium exchanger protein	G	T
	12	243316322	6.58	T	G	Genic	Mitochondrial proton/calcium exchanger protein	G	T
	12	243316335	6.58	G	A	Genic	Mitochondrial proton/calcium exchanger protein	A	G
	12	36410086	6.28	A	C	Genic	Gamete expressed protein 1	C	A

**Table 4:** List of SNPs (genic) with strong associations to carotenoid traits in a diverse pepper collection.

Traits	Chr.	Pos.	−log (*p*-Value)	Ref.	Alt	SNP Type	Protein Feature
α-carotene	1	69188981	6.32	A	G	Genic	E3 ubiquitin-protein ligase SINAT2
	2	139769588	6.64	C	T	Genic	Protein SCAR1 (AtSCAR1)
	3	257691563	7.83	C	T	Genic	Sucrose synthase 6 (AtSUS6)
	3	278759458	8.02	T	G	Genic	DENN domain and WD repeat-containing protein SCD1
	3	282407208	10.25	G	A	Genic	Hexokinase-1
	5	1431693	7.96	C	A	Genic	Putative disease resistance protein RGA1
	5	212795785	6.16	G	A	Genic	Ribosomal biogenesis protein LAS1L
	6	7784015	7.98	A	G	Genic	3-oxoacyl-[acyl-carrier-protein] synthase, mitochondrial
	6	152007017	7.93	G	A	Genic	Molybdenum cofactor sulfurase (MCS)
	6	236907247	9.77	A	T	Genic	U-box domain-containing protein 13
	7	193979854	6.07	A	G	Genic	Thymidylate kinase (AtTMPK)
	8	90513554	7.77	C	T	Genic	Mitochondrial carrier protein CoAc1
	8	139507673	9.86	T	C	Genic	Ribosome biogenesis protein bms1
	9	151061309	9.84	G	A	Genic	Protein SPA1-RELATED 3
	10	232860589	9.92	C	T	Genic	Disease resistance protein At4g27190,2
	11	253614532	9.84	C	A	Genic	Putative disease resistance RPP13-like protein 1
	12	37631709	9.79	A	T	Genic	Pentatricopeptide repeat-containing protein, chloroplastic
Antheraxanthin	1	1790608	7.04	G	A	Genic	Transcription termination factor MTEF18, mitochondrial
	1	13687346	6.76	A	T	Genic	tRNA N(3)-methylcytidine methyltransferase
	1	105823683	6.02	G	A	Genic	1:Probable methyltransferase PMT2
	4	1032836	5.97	C	T	Genic	Phosphatidylinositol 4-kinase alpha 1
	6	235401244	6.17	G	A	Genic	Protein disulfide isomerase-like 1–4
	6	235401309	6.16	G	A	Genic	Protein disulfide isomerase-like 1–4
	7	1570608	10.06	C	G	Genic	CA.PGAv.1.6.scaffold1357.17
	7	15180043	6.72	G	A	Genic	Chaperone protein DnaJ 2
	7	215963930	7.84	G	T	Genic	Probable NADH dehydrogenase [ubiquinone]
	8	139306996	6.65	C	A	Genic	Peptide-N(4)-(N-acetyl-beta-glucosaminyl)asparagine amidase
	10	980949	7.44	G	A	Genic	Septin and tuftelin-interacting protein 1 homolog 1
	11	251812831	6.06	G	A	Genic	Beta-glucosidase BoGH3B
	12	242740510	8.09	G	T	Genic	Disease resistance protein Roq1
	12	242740511	8.08	C	T	Genic	Disease resistance protein Roq1
β-carotene	6	125911050	6.61	A	G	Genic	Probably inactive leucine-rich repeat receptor-like protein kinase
	7	210903829	6.00	G	A	Genic	Probable beta-1,3-galactosyltransferase 2
β-cryptoxanthin	1	69188981	6.94	A	G	Genic	E3 ubiquitin-protein ligase SINAT2
	3	260332995	7.65	T	C	Genic	Chaperone protein dnaJ 49
	5	228154657	8.18	C	A	Genic	Protein-tyrosine-phosphatase MKP1
	6	92442789	7.99	C	T	Genic	Stamen-specific protein FIL1
	7	63490717	7.81	A	G	Genic	Rop guanine nucleotide exchange factor 14
	8	124720627	7.89	C	T	Genic	DEAD-box ATP-dependent RNA helicase 8
	12	160178786	7.87	G	A	Genic	Protein MHF1 homolog (AtMHF1)
Capsanthin	5	17846748	6.56	T	C	Genic	Lignin-forming anionic peroxidase
	5	32192112	6.08	G	C	Genic	Tobamovirus multiplication protein 3
	5	228154657	8.01	C	A	Genic	Protein-tyrosine-phosphatase MKP1
	6	92442789	6.10	C	T	Genic	Stamen-specific protein FIL1
	7	63490717	6.63	A	G	Genic	Rop guanine nucleotide exchange factor 14
	8	124720627	6.10	C	T	Genic	DEAD-box ATP-dependent RNA helicase 8
	12	160178786	6.24	G	A	Genic	Protein MHF1 homolog
Capsorubin	1	253857646	5.99	C	T	Genic	Alanine--tRNA ligase
	4	2341870	6.15	C	A	Genic	Receptor-like protein 19
Zeaxanthin	12	17593725	6.11	C	T	Genic	Pleiotropic drug resistance protein 2
Total Carotenoid	6	97491774	6.13	G	C	Genic	Acid phosphatase 1
	6	184116834	6.10	C	T	Genic	Histone–lysine N-methyltransferase

## Data Availability

Relevant data are included in both the manuscript and the Supplementary Files.

## Supplementary Materials

The following supporting information can be downloaded at: https://www.mdpi.com/article/10.3390/plants13182562/s1, Figure S1. Distribution of SNPs across the chromosomes of *Capsicum* from 306 accessions. This heatmap visualization represents SNP density, with data aggregated into 1 Mb windows. The color intensity indicates the local SNP concentration, highlighting their distribution patterns along the 12 pepper chromosomes; Figure S2. Quantile–Quantile (Q-Q) plot represent the association between fruit color and carotenoids. a: fruit color, b: α-carotene, c: antheraxanthin, d: β-carotene, e: β-cryptoxanthin, f: capsanthin, g: capsorubin, h: violaxanthin, i: zeaxanthin, and j: total carotenoids. *X*-axis represents the expected *p*-value (−log(*p*-value)) and *Y*-axis represents the observed *p*-value (−log(*p*-value)); Figure S3. Examples of different fruit colors observed in pepper accessions from the core collections; Table S1. List of significantly associated SNPs to carotenoids; Table S2. Accessions number, species name, fruit colors, and carotenoid contents of 306 pepper genetic resources.

## References

[1] Tripodi P., Kumar S., Ramchiary N., Kole C. (2019). The *Capsicum* Crop: An Introduction. The Capsicum Genome.

[2] Pereira-Dias L., Vilanova S., Fita A., Prohens J., Rodríguez-Burruezo A. (2019). Genetic Diversity, Population Structure, and Relationships in a Collection of Pepper (*Capsicum* spp.) Landraces from the Spanish Centre of Diversity Revealed by Genotyping-by-Sequencing (GBS). Hortic. Res..

[3] Sun T., Yuan H., Cao H., Yazdani M., Tadmor Y., Li L. (2018). Carotenoid Metabolism in Plants: The Role of Plastids. Mol. Plant.

[4] Antonio A.S., Wiedemann L.S.M., Veiga Junior V.F. (2018). The Genus *Capsicum*: A Phytochemical Review of Bioactive Secondary Metabolites. RSC Adv..

[5] Baenas N., Belović M., Ilic N., Moreno D.A., García-Viguera C. (2019). Industrial Use of Pepper (*Capsicum annum* L.) Derived Products: Technological Benefits and Biological Advantages. Food Chem..

[6] Borovsky Y., Tadmor Y., Bar E., Meir A., Lewinsohn E., Paran I. (2013). Induced Mutation in β-CAROTENE HYDROXYLASE Results in Accumulation of β-Carotene and Conversion of Red to Orange Color in Pepper Fruit. Theor. Appl. Genet..

[7] Tian S.-L., Li L., Shah S.N.M., Gong Z.-H. (2015). The Relationship between Red Fruit Colour Formation and Key Genes of Capsanthin Biosynthesis Pathway in *Capsicum annuum*. Biol. Plant.

[8] Venkatesh J., Lee S.-Y., Back S., Kim T.-G., Kim G.W., Kim J.-M., Kwon J.-K., Kang B.-C. (2023). Update on the Genetic and Molecular Regulation of the Biosynthetic Pathways Underlying Pepper Fruit Color and Pungency. Curr. Plant Biol..

[9] Guzman I., Hamby S., Romero J., Bosland P.W., O’Connell M.A. (2010). Variability of Carotenoid Biosynthesis in Orange Colored *Capsicum* spp.. Plant Sci..

[10] De Azevedo-Meleiro C.H., Rodriguez-Amaya D.B. (2009). Qualitative and Quantitative Differences in the Carotenoid Composition of Yellow and Red Peppers Determined by HPLC-DAD-MS. J. Sep. Sci..

[11] Liu Y. (2018). The Impacts of Artificial Intelligence on Design. Landsc. Archit. Front..

[12] Borovsky Y., Oren-Shamir M., Ovadia R., De Jong W., Paran I. (2004). The A Locus That Controls Anthocyanin Accumulation in Pepper Encodes a MYB Transcription Factor Homologous to Anthocyanin2 of Petunia. Theor. Appl. Genet..

[13] Lightbourn G.J., Griesbach R.J., Novotny J.A., Clevidence B.A., Rao D.D., Stommel J.R. (2008). Effects of Anthocyanin and Carotenoid Combinations on Foliage and Immature Fruit Color of *Capsicum annuum* L.. J. Hered..

[14] Berry H.M., Rickett D.V., Baxter C.J., Enfissi E.M.A., Fraser P.D. (2019). Carotenoid Biosynthesis and Sequestration in Red Chilli Pepper Fruit and Its Impact on Colour Intensity Traits. J. Exp. Bot..

[15] Krinsky N.I., Johnson E.J. (2005). Carotenoid Actions and Their Relation to Health and Disease. Mol. Asp. Med..

[16] Tanumihardjo S.A., Russell R.M., Stephensen C.B., Gannon B.M., Craft N.E., Haskell M.J., Lietz G., Schulze K., Raiten D.J. (2016). Biomarkers of Nutrition for Development (BOND)—Vitamin A Review. J. Nutr..

[17] Buscemi S., Corleo D., Di Pace F., Petroni M.L., Satriano A., Marchesini G. (2018). The Effect of Lutein on Eye and Extra-Eye Health. Nutrients.

[18] Story E.N., Kopec R.E., Schwartz S.J., Harris G.K. (2010). An Update on the Health Effects of Tomato Lycopene. Annu. Rev. Food Sci. Technol..

[19] Kotake-Nara E., Miyashita K., Nagao A., Kushiro M., Zhang H., Sugawara T. (2001). Carotenoids Affect Proliferation of Human Prostate Cancer Cells. J. Nutr..

[20] Arimboor R., Natarajan R.B., Menon K.R., Chandrasekhar L.P., Moorkoth V. (2015). Red Pepper (*Capsicum annuum*) Carotenoids as a Source of Natural Food Colors: Analysis and Stability—A Review. J. Food Sci. Technol..

[21] Fayos O., Ochoa-Alejo N., De La Vega O.M., Savirón M., Orduna J., Mallor C., Barbero G.F., Garcés-Claver A. (2019). Assessment of Capsaicinoid and Capsinoid Accumulation Patterns during Fruit Development in Three Chili Pepper Genotypes (*Capsicum* spp.) Carrying *Pun1* and *pAMT* Alleles Related to Pungency. J. Agric. Food Chem..

[22] Carvalho A.V., De Andrade Mattietto R., De Oliveira Rios A., De Almeida Maciel R., Moresco K.S., De Souza Oliveira T.C. (2015). Bioactive Compounds and Antioxidant Activity of Pepper (*Capsicum* sp.) Genotypes. J. Food Sci. Technol..

[23] Lefebvre V., Kuntz M., Camara B., Palloix A. (1998). The Capsanthin-Capsorubin Synthase Gene: A Candidate Gene for the y Locus Controlling the Red Fruit Colour in Pepper. Plant Mol. Biol..

[24] Huh J.H., Kang B.C., Nahm S.H., Kim S., Ha K.S., Lee M.H., Kim B.D. (2001). A Candidate Gene Approach Identified Phytoene Synthase as the Locus for Mature Fruit Color in Red Pepper (*Capsicum* spp.). Theor. Appl. Genet..

[25] Lang Y.-Q., Yanagawa S., Sasanuma T., Sasakuma T. (2004). Orange Fruit Color in *Capsicum* Due to Deletion of Capsanthin-Capsorubin Synthesis Gene. Breed. Sci..

[26] Hong M., Chi Z.-H., Wang Y.-Q., Tang Y.-M., Deng Q.-X., He M.-Y., Wang R.-K., He Y.-Z. (2019). Expression of a Chromoplast-Specific Lycopene β-Cyclase Gene (CYC-B) is Implicated in Carotenoid Accumulation and Coloration in the Loquat. Biomolecules.

[27] Han K., Jeong H.-J., Yang H.-B., Kang S.-M., Kwon J.-K., Kim S., Choi D., Kang B.-C. (2016). An Ultra-High-Density Bin Map Facilitates High-Throughput QTL Mapping of Horticultural Traits in Pepper (*Capsicum annuum*). DNA Res..

[28] Nimmakayala P., Abburi V.L., Saminathan T., Alaparthi S.B., Almeida A., Davenport B., Nadimi M., Davidson J., Tonapi K., Yadav L. (2016). Genome-Wide Diversity and Association Mapping for Capsaicinoids and Fruit Weight in *Capsicum annuum* L.. Sci. Rep..

[29] Wu L., Wang P., Wang Y., Cheng Q., Lu Q., Liu J., Li T., Ai Y., Yang W., Sun L. (2019). Genome-Wide Correlation of 36 Agronomic Traits in the 287 Pepper (*Capsicum*) Accessions Obtained from the SLAF-Seq-Based GWAS. Int. J. Mol. Sci..

[30] Fu G., Yu S., Wu K., Yang M., Altaf M.A., Wu Z., Deng Q., Lu X., Fu H., Wang Z. (2024). Genome-Wide Association Study and Candidate Gene Identification for Agronomic Traits in 182 Upward-Growing Fruits of *C. frutescens* and *C. annuum*. Sci. Rep..

[31] Huang X., Wei X., Sang T., Zhao Q., Feng Q., Zhao Y., Li C., Zhu C., Lu T., Zhang Z. (2010). Genome-Wide Association Studies of 14 Agronomic Traits in Rice Landraces. Nat. Genet..

[32] Zhao K., Tung C.-W., Eizenga G.C., Wright M.H., Ali M.L., Price A.H., Norton G.J., Islam M.R., Reynolds A., Mezey J. (2011). Genome-Wide Association Mapping Reveals a Rich Genetic Architecture of Complex Traits in Oryza Sativa. Nat. Commun..

[33] Li H., Peng Z., Yang X., Wang W., Fu J., Wang J., Han Y., Chai Y., Guo T., Yang N. (2013). Genome-Wide Association Study Dissects the Genetic Architecture of Oil Biosynthesis in Maize Kernels. Nat. Genet..

[34] Liu J., He Z., Rasheed A., Wen W., Yan J., Zhang P., Wan Y., Zhang Y., Xie C., Xia X. (2017). Genome-Wide Association Mapping of Black Point Reaction in Common Wheat (*Triticum aestivum* L.). BMC Plant Biol..

[35] Tian S.-L., Li L., Chai W.-G., Shah S.N.M., Gong Z.-H. (2014). Effects of Silencing Key Genes in the Capsanthin Biosynthetic Pathway on Fruit Color of Detached Pepper Fruits. BMC Plant Biol..

[36] Sharoni Y., Linnewiel-Hermoni K., Khanin M., Salman H., Veprik A., Danilenko M., Levy J. (2012). Carotenoids and Apocarotenoids in Cellular Signaling Related to Cancer: A Review. Mol. Nutr. Food Res..

[37] Sporn M.B., Liby K.T. (2013). Is Lycopene an Effective Agent for Preventing Prostate Cancer?. Cancer Prev. Res..

[38] Jeong H.-B., Jang S.-J., Kang M.-Y., Kim S., Kwon J.-K., Kang B.-C. (2020). Candidate Gene Analysis Reveals That the Fruit Color Locus C1 Corresponds to PRR2 in Pepper (*Capsicum frutescens*). Front. Plant Sci..

[39] Kahlau S., Bock R. (2008). Plastid Transcriptomics and Translatomics of Tomato Fruit Development and Chloroplast-to-Chromoplast Differentiation: Chromoplast Gene Expression Largely Serves the Production of a Single Protein. Plant Cell.

[40] Egea I., Barsan C., Bian W., Purgatto E., Latche A., Chervin C., Bouzayen M., Pech J.-C. (2010). Chromoplast Differentiation: Current Status and Perspectives. Plant Cell Physiol..

[41] Barsan C., Zouine M., Maza E., Bian W., Egea I., Rossignol M., Bouyssie D., Pichereaux C., Purgatto E., Bouzayen M. (2012). Proteomic Analysis of Chloroplast-to-Chromoplast Transition in Tomato Reveals Metabolic Shifts Coupled with Disrupted Thylakoid Biogenesis Machinery and Elevated Energy-Production Components. Plant Physiol..

[42] Suzuki M., Takahashi S., Kondo T., Dohra H., Ito Y., Kiriiwa Y., Hayashi M., Kamiya S., Kato M., Fujiwara M. (2015). Plastid Proteomic Analysis in Tomato Fruit Development. PLoS ONE.

[43] Haile M., Ro N., Ko H.-C., Oh H., Lee G.-A. (2023). A Comprehensive Genome-Wide Association Study of Carotenoid and Capsaicinoid Contents in *Capsicum chinense* Germplasm. Int. J. Mol. Sci..

[44] Ko H.-C., Haile M., Lee S., Hwang A., Lee G.-A., Choi Y.-M., Hahn B.-S., Ro N. (2022). Correlation of Carotenoids Content and ASTA Values of Pepper (*Capsicum chinense*) Genetic Resources. Horticulturae.

[45] Rodríguez-Rodríguez E., Sánchez-Prieto M., Olmedilla-Alonso B. (2020). Assessment of Carotenoid Concentrations in Red Peppers (*Capsicum annuum*) under Domestic Refrigeration for Three Weeks as Determined by HPLC-DAD. Food Chem. X.

[46] Bosland P.W., Janick J. (1996). *Capsicums*: Innovative Uses of an Ancient Crop. Progress in New Crops.

[47] Fernández-García E., Carvajal-Lérida I., Pérez-Gálvez A. (2016). Carotenoids Exclusively Synthesized in Red Pepper (Capsanthin and Capsorubin) Protect Human Dermal Fibroblasts against UVB Induced DNA Damage. Photochem. Photobiol. Sci..

[48] Jo S.J., Kim J.W., Choi H.O., Kim J.H., Kim H.J., Woo S.H., Han B.H. (2017). Capsanthin Inhibits Both Adipogenesis in 3T3-L1 Preadipocytes and Weight Gain in High-Fat Diet-Induced Obese Mice. Biomol. Ther..

[49] Mohd Hassan N., Yusof N.A., Yahaya A.F., Mohd Rozali N.N., Othman R. (2019). Carotenoids of *Capsicum* Fruits: Pigment Profile and Health-Promoting Functional Attributes. Antioxidants.

[50] Subburaj S., Tu L., Lee K., Park G.-S., Lee H., Chun J.-P., Lim Y.-P., Park M.-W., McGregor C., Lee G.-J. (2020). A Genome-Wide Analysis of the Pentatricopeptide Repeat (PPR) Gene Family and PPR-Derived Markers for Flesh Color in Watermelon (*Citrullus lanatus*). Genes.

[51] Park G., Shahwar D., Jang G., Shin J., Kwon G., Kim Y., Hong C.O., Jin B., Kim H., Lee O. (2023). Identification of a Novel Locus C2 Controlling Canary Yellow Flesh Color in Watermelons. Front. Genet..

[52] Galpaz N., Gonda I., Shem-Tov D., Barad O., Tzuri G., Lev S., Fei Z., Xu Y., Mao L., Jiao C. (2018). Deciphering Genetic Factors That Determine Melon Fruit-quality Traits Using RNA -Seq-based High-resolution QTL and eQTL Mapping. Plant J..

[53] Eriksson E.M., Bovy A., Manning K., Harrison L., Andrews J., De Silva J., Tucker G.A., Seymour G.B. (2004). Effect of the *Colorless Non-Ripening* Mutation on Cell Wall Biochemistry and Gene Expression during Tomato Fruit Development and Ripening. Plant Physiol..

[54] Williams P.M., Barkan A. (2003). A Chloroplast-localized PPR Protein Required for Plastid Ribosome Accumulation. Plant J..

[55] Wang X., An Y., Xu P., Xiao J. (2021). Functioning of PPR Proteins in Organelle RNA Metabolism and Chloroplast Biogenesis. Front. Plant Sci..

[56] Qin T., Zhao P., Sun J., Zhao Y., Zhang Y., Yang Q., Wang W., Chen Z., Mai T., Zou Y. (2021). Research Progress of PPR Proteins in RNA Editing, Stress Response, Plant Growth and Development. Front. Genet..

[57] Bhattarai A., Nimmakayala P., Davenport B., Natarajan P., Tonapi K., Kadiyala S.S., Lopez-Ortiz C., Ibarra-Muñoz L., Chakrabarti M., Benedito V. (2024). Genetic Tapestry of *Capsicum* Fruit Colors: A Comparative Analysis of Four Cultivated Species. Theor. Appl. Genet..

[58] Mol J., Grotewold E., Koes R. (1998). How Genes Paint Flowers and Seeds. Trends Plant Sci..

[59] Chalker-Scott L. (1999). Environmental Significance of Anthocyanins in Plant Stress Responses. Photochem. Photobiol..

[60] Mink P.J., Scrafford C.G., Barraj L.M., Harnack L., Hong C.-P., Nettleton J.A., Jacobs D.R. (2007). Flavonoid Intake and Cardiovascular Disease Mortality: A Prospective Study in Postmenopausal Women. Am. J. Clin. Nutr..

[61] Koes R., Verweij W., Quattrocchio F. (2005). Flavonoids: A Colorful Model for the Regulation and Evolution of Biochemical Pathways. Trends Plant Sci..

[62] Hichri I., Barrieu F., Bogs J., Kappel C., Delrot S., Lauvergeat V. (2011). Recent Advances in the Transcriptional Regulation of the Flavonoid Biosynthetic Pathway. J. Exp. Bot..

[63] Kitamura S., Shikazono N., Tanaka A. (2004). *TRANSPARENT TESTA 19* Is Involved in the Accumulation of Both Anthocyanins and Proanthocyanidins in *Arabidopsis*. Plant J..

[64] Marrs K.A., Alfenito M.R., Lloyd A.M., Walbot V. (1995). A Glutathione S-Transferase Involved in Vacuolar Transfer Encoded by the Maize Gene Bronze-2. Nature.

[65] Alfenito M.R., Souer E., Goodman C.D., Buell R., Mol J., Koes R., Walbot V. (1998). Functional Complementation of Anthocyanin Sequestration in the Vacuole by Widely Divergent Glutathione *S*-Transferases. Plant Cell.

[66] Sun Y., Li H., Huang J.-R. (2012). Arabidopsis TT19 Functions as a Carrier to Transport Anthocyanin from the Cytosol to Tonoplasts. Mol. Plant.

[67] Zhao J. (2015). Flavonoid Transport Mechanisms: How to Go, and with Whom. Trends Plant Sci..

[68] Hu B., Zhao J., Lai B., Qin Y., Wang H., Hu G. (2016). LcGST4 Is an Anthocyanin-Related Glutathione S-Transferase Gene in Litchi Chinensis Sonn. Plant Cell Rep..

[69] Pérez-Díaz R., Madrid-Espinoza J., Salinas-Cornejo J., González-Villanueva E., Ruiz-Lara S. (2016). Differential Roles for VviGST1, VviGST3, and VviGST4 in Proanthocyanidin and Anthocyanin Transport in Vitis Vinífera. Front. Plant Sci..

[70] Luo H., Dai C., Li Y., Feng J., Liu Z., Kang C. (2018). Reduced Anthocyanins in Petioles Codes for a GST Anthocyanin Transporter That Is Essential for the Foliage and Fruit Coloration in Strawberry. J. Exp. Bot..

[71] Jiang S., Chen M., He N., Chen X., Wang N., Sun Q., Zhang T., Xu H., Fang H., Wang Y. (2019). MdGSTF6, Activated by MdMYB1, Plays an Essential Role in Anthocyanin Accumulation in Apple. Hortic. Res..

[72] Welsch R., Maass D., Voegel T., DellaPenna D., Beyer P. (2007). Transcription Factor RAP2.2 and Its Interacting Partner SINAT2: Stable Elements in the Carotenogenesis of Arabidopsis Leaves. Plant Physiol..

[73] Xie Q., Guo H.-S., Dallman G., Fang S., Weissman A.M., Chua N.-H. (2002). SINAT5 Promotes Ubiquitin-Related Degradation of NAC1 to Attenuate Auxin Signals. Nature.

[74] Park B.S., Sang W.G., Yeu S.Y., Choi Y.D., Paek N.-C., Kim M.C., Song J.T., Seo H.S. (2007). Post-Translational Regulation of FLC Is Mediated by an E3 Ubiquitin Ligase Activity of SINAT5 in Arabidopsis. Plant Sci..

[75] Park B.S., Eo H.J., Jang I.-C., Kang H.-G., Song J.T., Seo H.S. (2010). Ubiquitination of LHY by SINAT5 Regulates Flowering Time and Is Inhibited by DET1. Biochem. Biophys. Res. Commun..

[76] Nolan T.M., Brennan B., Yang M., Chen J., Zhang M., Li Z., Wang X., Bassham D.C., Walley J., Yin Y. (2017). Selective Autophagy of BES1 Mediated by DSK2 Balances Plant Growth and Survival. Dev. Cell.

[77] Wang W., Fan Y., Niu X., Miao M., Kud J., Zhou B., Zeng L., Liu Y., Xiao F. (2018). Functional Analysis of the Seven in Absentia Ubiquitin Ligase Family in Tomato. Plant Cell Environ..

[78] Qi H., Xia F.-N., Xie L.-J., Yu L.-J., Chen Q.-F., Zhuang X.-H., Wang Q., Li F., Jiang L., Xie Q. (2017). TRAF Family Proteins Regulate Autophagy Dynamics by Modulating AUTOPHAGY PROTEIN6 Stability in Arabidopsis. Plant Cell.

[79] Zhang C., Hao Z., Ning Y., Wang G.-L. (2019). SINA E3 Ubiquitin Ligases: Versatile Moderators of Plant Growth and Stress Response. Mol. Plant.

[80] Fu J., Wu Q., Wang X., Sun J., Liao L., Li L., Xu Q. (2024). A Novel Histone Methyltransferase Gene CgSDG40 Positively Regulates Carotenoid Biosynthesis during Citrus Fruit Ripening1. J. Integr. Agric..

[81] Cazzonelli C.I., Cuttriss A.J., Cossetto S.B., Pye W., Crisp P., Whelan J., Finnegan E.J., Turnbull C., Pogson B.J. (2009). Regulation of Carotenoid Composition and Shoot Branching in *Arabidopsis* by a Chromatin Modifying Histone Methyltransferase, SDG8. Plant Cell.

[82] Liu S., Liu M., Cao Y., Xu Y., Liu H., Zhu Q., Zhang X., Luan F. (2023). Identification of Chromosome Region and Candidate Genes for Canary-Yellow Flesh (Cyf) Locus in Watermelon (*Citrullus lanatus*). Plant Sci..

[83] Zhang J., Sun H., Guo S., Ren Y., Li M., Wang J., Yu Y., Zhang H., Gong G., He H. (2022). ClZISO Mutation Leads to Photosensitive Flesh in Watermelon. Theor. Appl. Genet..

[84] Kim J.K., Lee S.Y., Chu S.M., Lim S.H., Suh S.-C., Lee Y.-T., Cho H.S., Ha S.-H. (2010). Variation and Correlation Analysis of Flavonoids and Carotenoids in Korean Pigmented Rice (*Oryza sativa* L.) Cultivars. J. Agric. Food Chem..

[85] Lee H.-Y., Ro N.-Y., Jeong H.-J., Kwon J.-K., Jo J., Ha Y., Jung A., Han J.-W., Venkatesh J., Kang B.-C. (2016). Genetic Diversity and Population Structure Analysis to Construct a Core Collection from a Large *Capsicum* Germplasm. BMC Genet..

[86] Elshire R.J., Glaubitz J.C., Sun Q., Poland J.A., Kawamoto K., Buckler E.S., Mitchell S.E. (2011). A Robust, Simple Genotyping-by-Sequencing (GBS) Approach for High Diversity Species. PLoS ONE.

[87] De Donato M., Peters S.O., Mitchell S.E., Hussain T., Imumorin I.G. (2013). Genotyping-by-Sequencing (GBS): A Novel, Efficient and Cost-Effective Genotyping Method for Cattle Using Next-Generation Sequencing. PLoS ONE.

[88] Catchen J., Hohenlohe P.A., Bassham S., Amores A., Cresko W.A. (2013). Stacks: An Analysis Tool Set for Population Genomics. Mol. Ecol..

[89] Andrews S. (2010). A Quality Control Tool for High Throughput Sequence Data. http://www.bioinformatics.babraham.ac.uk/projects/fastqc/.

[90] Martin M. (2011). Cutadapt Removes Adapter Sequences from High-Throughput Sequencing Reads. EMBnet J..

[91] Bradbury P.J., Zhang Z., Kroon D.E., Casstevens T.M., Ramdoss Y., Buckler E.S. (2007). TASSEL: Software for Association Mapping of Complex Traits in Diverse Samples. Bioinformatics.

