# Supplementary material for: Genome-Wide Analysis of Fruit Color and Carotenoid Content in Capsicum Core Collection
Source: Plants (Basel). 2024 Sep 12;13(18):2562. doi: 10.3390/plants13182562 (PMC11435234; doi:10.3390/plants13182562)
Supplement: Supplementary file 1 [file plants-13-02562-s001.zip › Supplementary Figures.pdf]

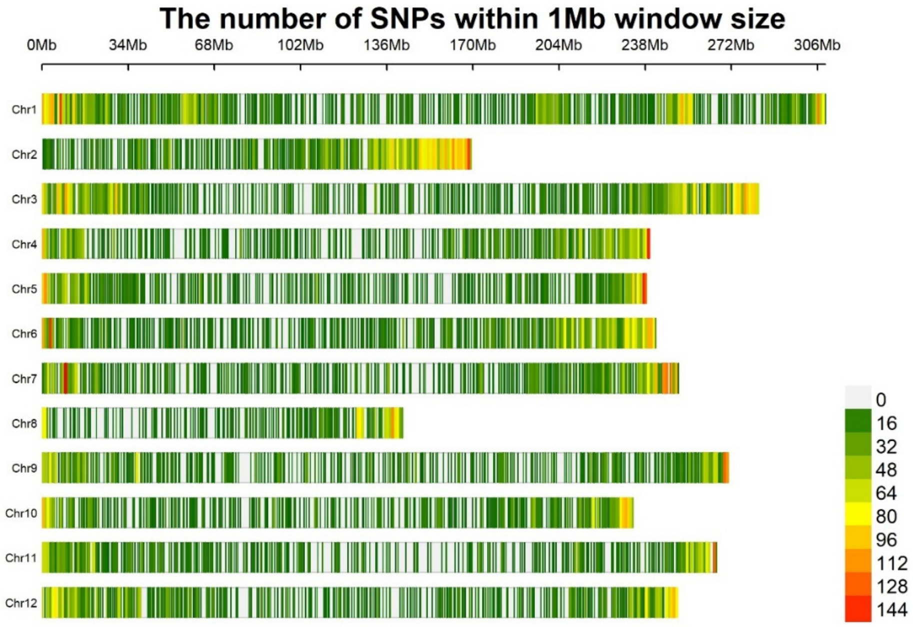

Supplementary Figure S1. Distribution of SNPs across the chromosomes of *Capsicum* from 306 accessions. This heatmap visualization represents SNP density, with data aggregated into 1 Mb windows. The color intensity indicates the local SNP concentration, highlighting their distribution patterns along the 12 pepper chromosomes.

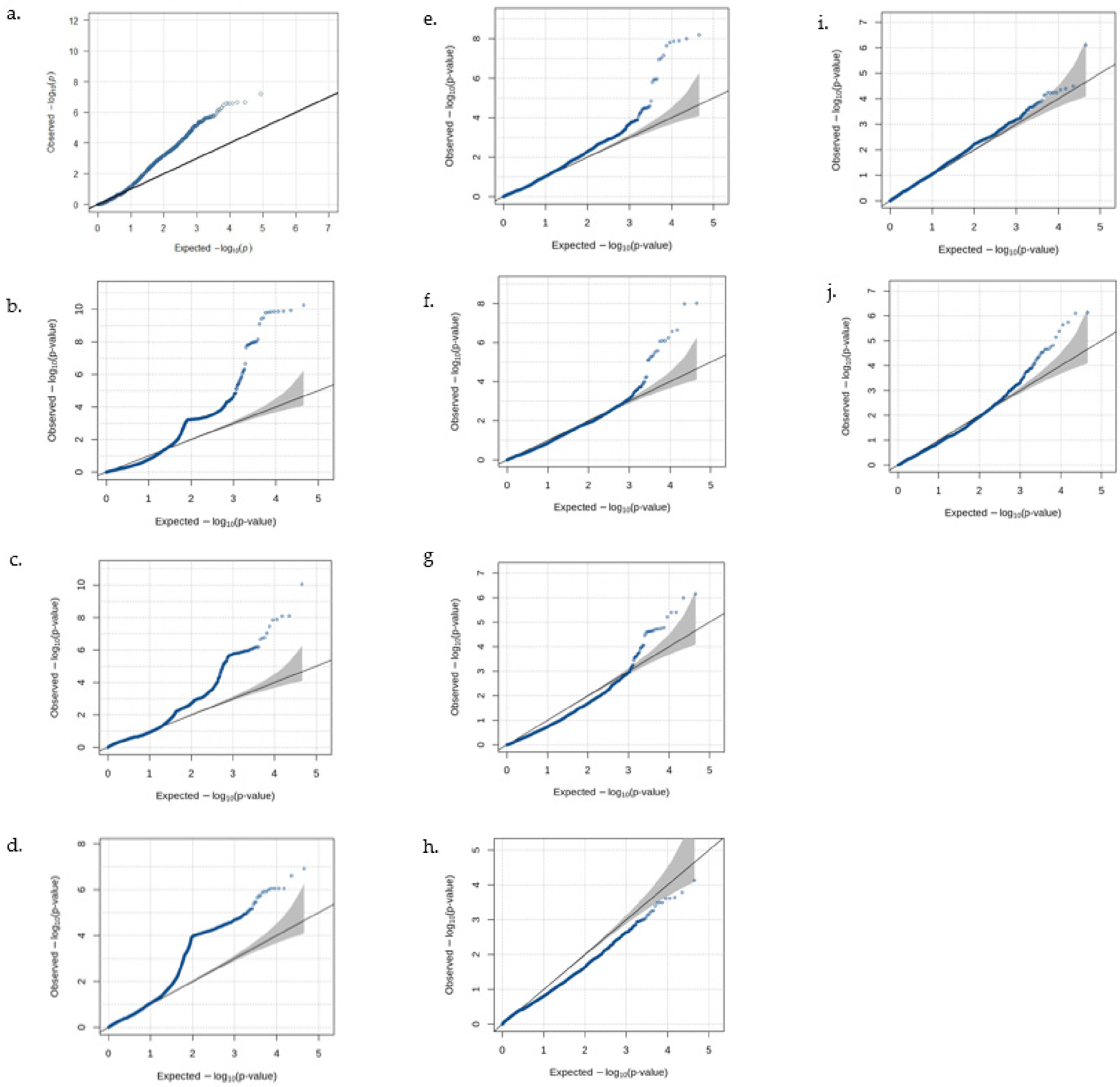

Supplementary Figure S2. Quantile-Quantile (Q-Q) plot represent the association between fruit color and carotenoids. a: fruit color , b: *α*-carotene, c: antheraxanthin, d: β-carotene, e: β-cryptoxanthin, f: capsanthin, g: capsorubin, h: violaxanthin, i: zeaxanthin and j: total carotenoids. X-axis represent the expected *p*-value (-log(*p*-value)) and Y-axis represents the observed *p*-value (-log(*p*-value)).

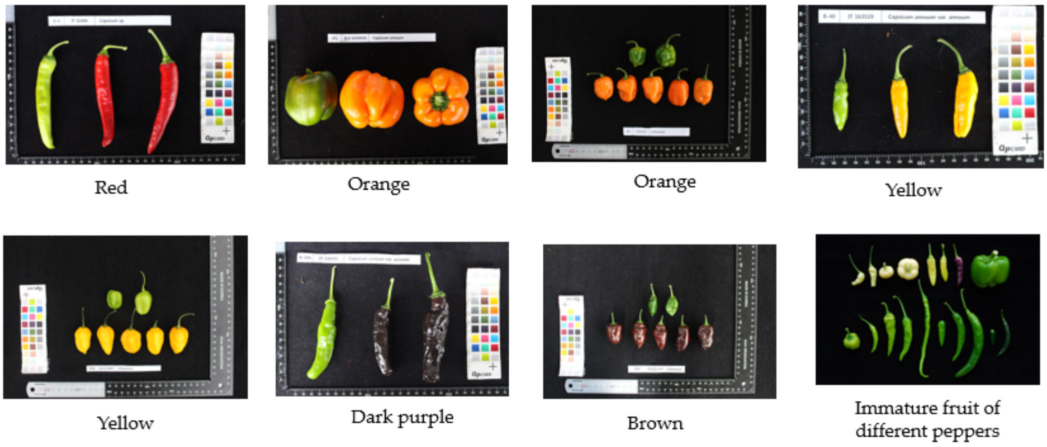

Supplementary Figure S3. Examples of different fruit colors observed in pepper accessions at maturity stage from the core collections.
